# The determinants of knowledge of cervical cancer, attitude towards screening and practice of cervical cancer prevention amongst antenatal attendees in Ibadan, Southwest Nigeria

**DOI:** 10.3332/ecancer.2021.1225

**Published:** 2021-05-05

**Authors:** Adebayo M D Agboola, Oluwasomidoyin O Bello

**Affiliations:** 1 Department of Obstetrics and Gynaecology, University College Hospital, University of Ibadan, PMB 5116, Ibadan, Oyo State, Nigeria; 2 Department of Obstetrics and Gynaecology, College of Medicine, University College Hospital, University of Ibadan, PMB 5116, Ibadan, Oyo State, Nigeria

**Keywords:** cervical cancer, antenatal attendees, Pap smear, cervical cancer screening, cervical cancer prevention, Nigeria

## Abstract

Cervical cancer (CC) is an extremely preventable and curable disease with early detection and treatment. Unfortunately, the practice of cervical cancer prevention (CCP) remains poor in resource constrained countries. This study aimed to identify determinants of knowledge of CC, attitude towards cervical cancer screening (CCS) and practice of CCP among antenatal attendees in a tertiary hospital in Southwest Nigeria as they are sexually active women. This was a cross-sectional survey of a cohort of 287 antenatal attendees using a self-administered structured questionnaire to assess their knowledge of CC, attitude towards screening and uptake of screening and human papillomaviruses (HPVs) vaccination as methods of practice of CCP. Data was analysed using the Statistical Package for Social Sciences version 20.0. Descriptive statistics were conducted for all relevant data. Categorical variables were explored using chi-square test and the independent variables with significant associations (*p*-value < 0.05) entered into logistic regression analysis. The mean age was 30.62 ± 4.5 years. Three-fifths (60.6%) of the women had good knowledge of CC while 47.4% had heard about CCS. Majority (75.6%) were willing to undergo CCS thereby exhibiting positive attitude towards screening. The practice of CCP was poor as only 27 (9.4%) had ever been screened for CC while 10 (3.5%) had received the HPV vaccine. Interestingly, none of the women who had received the HPV vaccine had been screened for CC. Those with tertiary education were more likely (OR = 2.140, 95% CI = 1.166–4.979) to exhibit positive attitude to CCS, while those with poor knowledge were about two times less likely to have a positive attitude (OR = 0.532, 95% CI = 0.291–0.972). Poor knowledge of CC was associated with lesser odds (OR = 0.061, 95% CI = 0.008–0.471) of practice of CCP. In Nigeria, the burden of CC can be reduced if women are educated and health care providers challenged to recommend CCS and HPV vaccination.

## Background

Cervical cancer (CC) is the most common gynaecological cancer. Ninety-nine percent of all cases are linked to infection with high-risk human papillomaviruses (HPVs) which may be transmitted through sexual contact [1]. CC ranks fourth among the common cancers in women worldwide; only breast, colorectal and lung cancers are more common [2]. Of the 570,000 cases of CC diagnosed globally in 2018, there were 311,000 deaths (85% of which were from developing countries). This makes it a major public health problem with a predisposition for middle-aged women in resource constrained countries [2]. Most of these deaths are largely preventable with access to comprehensive cervical cancer prevention (CCP) and control programmes which improve uptake of HPV vaccination among young girls, screening all at-risk women and treating pre-cancerous lesions [3].

The incidence of CC is disproportionately increased in developing countries. In Nigeria, 53.3 million women are estimated to be at risk of developing CC with a national standardised prevalence rate of 33.0 per 100,000 [4]. Many factors such as sexually transmitted infections, reproductive factors, hormonal influences, genetic and host factors have been implicated in the occurrence of CC. Complications associated with CC arise due to late reporting, ignorance and cultural issues relating to cervical cancer screening (CCS) [5, 6].

Regular screening has been shown to effectively detect early disease. Furthermore, the HPV vaccination has also contributed to a decline in the incidence rate of CC. However, several studies have reported that the awareness and uptake of CCS services has remained poor in developing countries despite its availability [6–8]. Provider collected or self-collected HPV testing, cytology-based Papanicolaou smear (Pap smear) and visual inspection with acetic acid (VIA) are common methods of CCS. Self-collected HPV testing and VIA are the most cost-effective and should be made readily available in low and middle income countries (LMICs) [9–11].

Inadequate knowledge about CC, lack of familiarity with the concept of prevention, the geographical and economic inaccessibility of care, the poor quality of services and lack of support from husbands and relatives have been identified as major hindrances to CCS [12]. Interestingly, studies in LMICs have shown that knowledge and awareness alone do not necessarily influence uptake of CCS [13–15]. In Nigeria, for instance, despite many campaigns, CCS modalities are hardly utilised [14, 15].

Pregnant women are a sexually active cohort who should ideally have been integrated into CCS schedules in most cases. For those who either have not had any or have a deficient screening history, studies done in North America have shown that pregnant women are still eligible for screening [16, 17]. Health talks on CC during antenatal visits can help bridge the communication gap that is responsible for the poor knowledge of CC and CCP. So far in Nigeria, there are few studies that have assessed the knowledge of CC and attitude towards its screening among pregnant women alone [18]. Most of the previous studies were either hospital or community-based surveys of non-pregnant women [6, 15, 19]. Interviewing pregnant women in antenatal clinics is an opportunity to access a representative sample of sexually active women. Therefore, this study aims to assess the determinants of knowledge of CC among antenatal attendees, their attitude towards CCS and practice of screening and HPV vaccination as methods to prevent CC.

## Methods

### Study setting and design

This was a facility-based cross-sectional study among pregnant women recruited from the antenatal clinic of the University College Hospital, Ibadan, Oyo State. This Tertiary centre provides care to about 3,000 pregnant women annually.

### Study population and eligibility criteria

The study enrolled consenting pregnant women between the ages of 18 and 45 years by simple random sampling. Women who presented with obstetric emergencies or did not give their consent were excluded from the study.

### Sample size

The sample size was calculated using a single population proportion formula. Using a 27.7% proportion (the number of correspondents who has adequate knowledge of CC in a similar study) [20], 95% confidence interval, 5% margin of error and an annual population of 3,000 antenatal attendees, the minimum sample size of 276 were derived. Using a non-response rate of 10%, a final sample size of 303 was used.

### Data collection

A self-administered, pre-tested structured questionnaire adapted from a similar study [20] was used to obtain information about the sociodemographic characteristics, knowledge of risk factors, symptoms and prevention of CC, attitude towards CCS and uptake among the women.

### Statistical analysis

The data was analysed using the Statistical Package for Social Sciences (SPSS) version 21 (Released 2012. IBM SPSS Statistics for Windows, Version 21.0, Armonk, NY: IBM Corp). Descriptive statistics were conducted for all relevant data. Categorical variables were explored using chi-square test and the independent variables with significant associations (*p*-value < 0.05) entered into logistic regression analysis to determine factors associated with knowledge of CC, attitude towards CCS and practice of CCP. Adopting a similar scoring system used in assessing CC knowledge [20], each correctly identified symptom or risk factor was assigned a score of 1, and a score of zero was assigned to an incorrect or ‘I don’t know’ response. The maximum obtainable score for CC knowledge was 25. Using the adopted scoring system [20], women who scored less than or equal to 13 were regarded as having poor knowledge while those who scored over 13 were considered as having good knowledge. Positive attitude towards screening was defined as willingness of the women to be screened for the CC while good practice of CCP was determined if they had undergone the screening for CC and/or received HPV vaccine.

### Ethical considerations

Ethical approval was obtained from the University of Ibadan (UI)/University College Hospital (UCH) Ethics committee with assigned number UI/EC/20/0256.

## Results

Three hundred and three pregnant women participated in the study; however, 287 responses were adequate giving a response rate of 94.7%. Their mean age was 30.62 ± 4.5 years and 65.9% were between 26 and 35 years. Majority (83.3%) had a tertiary level of education and about half (49.8%) were employed (Table 1).

Three-fifths (60.6%) of the women had good knowledge of CC while 58.0% reported it to be a common malignancy. Health workers were the most common (49.4%) source of information. Although about half (47.4%) of the women had heard about CCS, a higher proportion (75.6%) were willing to undergo CCS thereby exhibiting positive attitude towards screening. Only 48 (16.7%) women reported that they are susceptible to CC. For the others, some of the reasons for the non-susceptibility were ‘I have only one sexual partner’ in 95 (39.7%) and ‘I am spiritually protected’ in 39 (16.3%) (Table 2).

About 136 (47.4%) had heard about CCS but only 27 (9.4%) ever had a form of screening in the past. Pap smear was the most common (70.4%) screening modality. The main reasons for undergoing screening were a ‘health providers request’ (18.5%) or because ‘it was free’ (22.2%). Of the 90.6% who never had any form of CCS, 33.8% reported that it was because ‘health provider did not request for it’, 25% because they were ‘not experiencing symptoms’ and 11.9% thought ‘I cannot have cervical cancer’. Seventy-two women (25%) had heard of HPV vaccination and 10 (3.5%) had received it previously. None of the women who had received HPV vaccine had been screened for CC. Of the 25 women who had a child above 9 years, only 1 (4%) child had received the HPV vaccine. Only 2 in 10 women knew the HPV vaccine could be received by both boys and girls. One hundred and sixty-four (57.1%) women were willing to receive the HPV vaccine or recommend it to family and friends (Table 2).

Age (*p* = 0.015) and level of education (*p* = 0.029) were associated with good knowledge of CC. None of the demographic characteristics was associated with increased odds of having good knowledge of CC on multivariate regression analysis (Table 3).

The level of education (*p* = 0.001) and knowledge of CC (*p* = 0.003) were associated with the women’s attitude towards screening while knowledge of CC (*p* < 0.001) was the only significant factor associated with their practice of CCP (Table 4).

The women with tertiary education were more likely (OR = 2.140, 95% CI = 1.166–4.979) to have a positive attitude towards screening than those with ‘secondary or lower’ level of education. While those with poor knowledge of the disease had lower odds (OR = 0.532, 95% CI = 0.291–0.972) of having a positive attitude towards screening compared to those with good knowledge. Knowledge of CC was the only determinant of the practice of CCP. The women who had poor knowledge of the disease were less likely (OR = 0.061, 95% CI = 0.008–0.471) to practice CCP than those with good knowledge (Table 5).

## Discussion

This study is one of the few that have surveyed antenatal attendees on CC and CCP. About two-thirds exhibited good knowledge of CC in this study and this was a contrast to the poor knowledge recorded among participants in community based and hospital based studies in Africa [6–8, 21–23]. In a similar survey of antenatal attendees in South east Nigeria, the women were identified as having poor knowledge of CC [24]. Our finding may not be unconnected to the fact that a greater proportion of women in our study had tertiary level of education. Information about this disease was mostly through health workers, a finding which corroborates the reports from a hospital based survey of pregnant and non-pregnant women in Lagos, Nigeria [21]. However, the media was the main source of information in a community based survey of rural women also in Lagos, Nigeria [25]. Quality sources of information are necessary to nullify the many myths African women have about CC [25]. In a qualitative cross-sectional study in Northern Nigeria, the knowledge about the aetiology and risk factors of CC was poor among participants albeit with adequate awareness of the disease. The women thought the use of herbal concoctions and poor personal and environmental hygiene were the risk factors associated with CC [26]. Our study showed that age and level of education were associated with knowledge levels. However, multivariate analysis revealed that none of the demographic factors were associated with increased odds of having good knowledge of CC.

Most of the women surveyed did not consider themselves at risk for CC. The belief that non-susceptibility is due to spiritual protection is extremely flawed but not new [27]. If women feel shielded by their spirituality, then it means their understanding of the aetiology of the disease is poor and this may mitigate against CCP. Non-susceptibility is also reported by majority of the women of reproductive age in the southern region of the country who however acknowledge that CCS is beneficial [28]. Despite poor uptake, an overwhelming majority believe that CCS is important [29].

Although half of the respondents in our study were aware of CCS, only 1 in 10 of the women had ever been screened. The Pap smear was the most commonly used form of CCS while the combination of Pap smear and HPV testing was the least utilised. In Nigeria, the proportion of women who have been screened for CC has been consistently low [25]. These poor indices have been recorded in both rural and urban areas, across various geographical locations. However, in some of the studies where the awareness levels were high, it did not translate to adequate knowledge levels or uptake of CCS [21, 26, 30, 31]. A Pap smear is a highly sensitive and specific method that has been used for decades to detect pre-cancerous lesions of cervix [32]. The World Health Organization recommends the screen-and-treat approach with the preferred screening modality to include HPV testing except in environments without enough resources to test for the virus. In these areas, VIA is more cost-effective and treatment should be arranged at the same contact [33]. Another advantage of this screen-and-treat approach is that it reduces the incidence of loss to follow-up.

The women studied in our centre had not been screened for CC mainly because no health provider requested for it. Health providers played a vital role in promoting uptake of screening modalities among those who had been screened in the past. This pattern has also been documented in other studies [21, 34]. Health providers are the major influencers of the knowledge of CC and the uptake of CCS, while negative behaviours by health providers towards at-risk women have been described as a deterrent to the uptake of CCS [35]. These workers should be trained consistently to deliver health talks to individuals, groups or mass media and to perform thorough physical examinations so as to identify at-risk women and provide screening modalities [23]. The fact that a quarter of the respondents felt they did not need screening because they had no symptoms also implies that they do not understand the concept of CCP, the insidious nature of the disease and the fact that overt malignancy can be quite devastating. It is surprising that another group of women just concluded that they could not have CC even with no scientific evidence.

Majority of the women were willing to be screened for CC. This finding is in keeping with various reports in Nigeria [21, 25, 34, 36]. Multivariate analysis revealed that the willingness to undergo screening in our study was associated with knowledge of the disease and education status. Those with good knowledge of the disease were two times more likely to exhibit willingness for screening compared to those with poor knowledge while the women with tertiary education were more than two times more likely to be willing to be screened compared to those with secondary education or less. Other studies have shown that women in Southwest Nigeria are willing to undergo CCS [21, 25].

There was a poor uptake of Pap smear and HPV vaccine among the women surveyed. Those with good knowledge were about 16 times more likely to practice either form of CCP as against women with poor knowledge. Nigeria has a remarkably low level of uptake of Pap smear and HPV vaccination; [29, 37, 38] other African countries are not quite different [13, 39, 40]. It is important to increase the awareness of CCP in order to promote its practice [31]. Furthermore, the government has a role to play by establishing organised screening centres and adopting protocols that improve uptake [37]. Factors like inability to afford the cost of screening, not knowing where and how to access screening services, nonchalant attitude to one’s health are some reasons identified for poor uptake of screening [5].

Of the 25 women who had children above 9 years of age, only one woman reported her daughter had received the HPV vaccine and thus the burden of CC on the next generation can be implied. Studies across Africa have shown that many women and young girls are not receiving the HPV vaccine [11, 41]. Some factors like fear, cost of vaccine and lack of multiple sexual partners or lack of sexual activity have been identified as barriers to the uptake of HPV vaccine [41]. The willingness to receive HPV vaccine recorded in a little over half of the respondents is also suboptimal and may be a reflection of myths associated with vaccination. The fact that many more women in our study were willing to receive HPV vaccine if it was free signifies that cost may prevent some from receiving the vaccine.

While pregnant women are obviously at risk for CC, there is a paucity of studies regarding this subject matter among them. This group of women is accessible and they can be surveyed as an indirect measure of CCS in Nigerian women. This study did not assess if the women had received prior information/education pertaining CC and its screening during previous antenatal clinic visits. This aspect can be considered when designing similar research protocols in future studies. Also, a mixed methods approach to include one-on-one interviews may elicit myths or misconceptions about CC and other barriers to prevention.

## Conclusion

Many of the women surveyed demonstrated good knowledge of CC. However, the uptake of CCS and vaccination against HPV were grossly inadequate. Tertiary education and good knowledge of CC were determinants of positive attitude towards CCS while good knowledge of the disease influenced the practice of both screening and HPV vaccination as methods to prevent CC.

In Nigeria, the burden of CC can be reduced if women are educated and health care providers challenged to recommend CCS and HPV vaccination.

## Conflicts of interest

The authors declare that they have no competing interests.

## Funding

None.

## Figures and Tables

**Table 1.** Sociodemograpic characteristics of the antenatal attendees.

Variables	Frequency (%) *n* = 287
**Age (years)** ≤25 26–35 >35 Mean ± SD	39 (13.6) 189 (65.9) 59 (20.6) 30.62 ± 4.5
**Marital status** Single Married	21 (7.3) 266 (92.7)
**Ethnicity** Yoruba Igbo Hausa Others	245 (85.4) 26 (9.1) 3 (1.0) 13 (4.5)
**Level of education** None Primary Secondary Tertiary	3 (1.0) 2 (0.7) 43 (15.0) 239 (83.3)
**Employment status** Employed Self-employed Unemployed	143 (49.8) 112 (39.0) 32 (11.1)
**Religion** Christianity Islam	210 (73.2) 77 (26.8)

**Table 2.** Attitude towards CCP and uptake of screening and HPV vaccination.

Items	Frequency (%)
Are you susceptible to cancer of the cervix? (*n* = 287)	48 (16.7)
**Reasons for assuming non-susceptibility (*n* = 239)**	
I have only a sexual partner	95 (39.7)
I am immune	14 (5.9)
I am spiritually protected	39 (16.3)
I don’t know	87 (36.4)
**Ever had screening for CC**	27 (9.4)
**Which test was done? (*n* = 27)**	
Pap smear	19 (70.4)
HPV test	3 (11.1)
Visual inspection test	4 (14.8)
Pap smear and HPV test	1 (3.7)
**How many times have you done the test?**	
1	19 (70.4)
2	7 (25.9)
4	1 (3.7)
**Reasons for having the screening test (*n* = 27)**	
Health providers request	5 (18.5)
It was free	6 (22.2)
Part of a general screening	3 (11.1)
Felt like doing it	13 (48.1)
**Reasons for not having the screening test (*n* = 260)**	
Unaware of the screening availability	44 (16.9)
Unaware of facilities rendering the service	17 (6.5)
No experiencing symptoms	65 (25.0)
Feel it is expensive	19 (7.3)
Health provider did not request for it	88 (33.8)
Never thought of it	52 (20.0)
I cannot have CC	31 (11.9)
I need my husband’s approval	9 (3.5)
**Ever received the HPV vaccine?**	10 (3.5)
**Who recommended it?**	
Health practitioner	8 (80.0)
Self	2 (20.0)
**Have a child above 9 years who has received the HPV vaccine (*n* = 25)**	1 (4.0)
**Willing to undergo CCS**	217 (75.6)
**Willing to receive the HPV vaccine or recommend it to family and friends**	164 (57.1)
**Will accept the vaccine if it is free**	253 (88.2)
**Will have a Pap smear if it is free**	253 (88.2)
**Who should receive the HPV vaccine?**	
Girls	219 (76.3)
Boys	4 (1.4)
Both	52 (18.1)
Nobody	12 (4.2)

**Table 3.** Bivariate and multivariate analysis of respondents’ characteristics with knowledge of CC.

Variables	Knowledge of CC		X^2^, *p*-value	OR (95% CI)	*p*-value
	Poor	Good			
**Age (years)**					
<25	7 (6.2)	32 (18.3)	8.43, 0.015	4.220 (0.646–27.545)	0.133
26–35	80 (71.4)	109 (62.3)		3.163 (0.652–15.346)	0.153
>35	25 (22.3)	34 (19.4)		1	
**Marital status**					
Married	104 (92.9)	162 (92.6)	0.008, 1.000	0.449 (0.143–1.415)	0.172
Single	8 (7.1)	13 (7.4)		1	
**Level of education**					
Tertiary	100 (89.3)	139 (79.4)	4.76, 0.029	0.148 (0.016–1.366)	0.092
Secondary or lower	12 (10.7)	36 (20.6)		1	
**Employment status**					
Unemployed	9 (8.0)	23 (13.1)	2.74, 0.254	0.588 (0.242–1.428)	0.241
Self-employed	49 (43.8)	63 (36.0)		0.574 (0.232–1.423)	0.231
Employed	54 (48.2)	89 (50.9)		1	
**Number of antenatal visits**					
Less or equal to 4	70 (62.5)	114 (65.1)	0.21, 0.649	1.184 (0.690–2.032)	0.540
More than 4	42 (37.5)	61 (34.9)		1	
**Perceived susceptibility to CC**					
Yes	16 (14.3)	32 (18.3)	0.79, 0.376	1.343 (0.699–2.581)	0.377
No	96 (85.7)	143 (81.7)		1	

**Table 4.** Bivariate analysis of respondents’ characteristics with attitude towards CCS and practice of CCP.

	Attitude towards CCS		X^2^, *p*-value	Practice of CCP			X^2^, *p*-value
	Negative	Positive		Yes	No		
**Age (years)**							
<25	24 (21.2)	15 (8.6)	10.91, 0.115	3 (8.1)	36 (14.4)		4.12, 0.128
26–35	72 (63.7)	117 (67.2)		22 (59.5)	167 (66.8)		
>35	17 (15.0)	42 (24.1)		12 (32.4)	47 (18.8)		
**Marital status**							
Married	8 (11.4)	13 (6.0)	2.31, 0.129	35 (94.6)	231 (92.4)		0.23, 1.000
Single	62 (88.6)	204 (94.0)		2 (5.4)	19 (7.6)		
**Level of education**							
Tertiary	21 (30.0)	27 (12.4)	11.72, 0.001	34 (91.9)	205 (82.0)		2.26, 0.132
Secondary or lower	49 (70.0)	190 (87.6)		3 (8.1)	45 (18.0)		
**Employment status**							
Unemployed	11 (15.7)	21 (9.7)	2.02, 0.364	4 (10.8)	28 (11.2)		1.74, 0.420
Self-employed	25 (35.7)	87 (40.1)		11 (29.7)	101 (40.4)		
Employed	34 (48.6)	109 (50.2)		22 (59.5)	121 (48.4)		
**Number of antenatal visits**							
Less or equal to 4	48 (68.6)	136 (62.7)	0.80, 0.371	28 (75.7)		156 (62.4)	2.47, 0.116
More than 4	22 (31.4)	81 (37.3)		9 (24.3)		94 (37.6)	
**Knowledge of CC**							
Poor	38 (54.3)	75 (34.6)	8.625, 0.003	4 (10.8)		109 (43.6)	14.52, <0.001
Good	32 (45.7)	142 (65.4)		33 (89.2)		141 (56.4)	

**Table 5.** Multivariate analysis of characteristics against attitude towards CCS and practice of CCP.

	Positive attitude to CCS		Practice of CCP	
	OR (95% CI)	*p*-value	OR (95% CI)	*p*-value
**Age (years)**				
<25	0.791 (0.302–2.069)	0.632	4.656 (0.466–46.702)	0.191
26–35	0.564 (0.28–1.124)	0.104	1.339 (0.525–3.417)	0.541
>35	1		1	
**Marital status**				
Married	1.545 (0.555–4.295)	0.405	0.798 (0.145–4.38)	0.795
Single	1		1	
**Level of education**				
Tertiary	2.410 (1.166–4.979)	0.018	2.747 (0.308–24.534)	0.366
Secondary or lower	1		1	
**Employment status**				
Unemployed	0.911 (0.356–2.334)	0.847	0.581 (0.205–4.310)	0.462
Self-employed	0.656 (0.252–1.711)	0.389	0.337 (0.089–1.280)	0.170
Employed	1		1	
**Number of antenatal visits**				
Less or equal to 4	1.270 (0.695–2.319)	0.437	0.611 (0.246–1.521)	0.290
More than 4	1		1	
**Knowledge of CC**				
Poor	0.532 (0.291–0.972)	0.040	0.061 (0.008–0.471)	0.007
Good	1		1	
